# The effect of pomegranate seed oil on human health, especially epidemiology of polycystic ovary syndrome; a systematic review

**DOI:** 10.5935/1518-0557.20210121

**Published:** 2022

**Authors:** Mahmoud Bahmani, Samira Shokri, Zabih Nosrati Akhtar, Saber Abbaszadeh, Aliasghar Manouchehri

**Affiliations:** 1 Biotechnology and Medicinal Plants Research Center, Ilam University of Medical Sciences, Ilam, Iran; 2 Department of Environmental Health Engineering, Division of Food Safety & Hygiene, School of Public Health, Tehran University of Medical Sciences, Tehran, Iran; 3 Boroujerd Branch, Islamic Azad University, Boroujerd,Iran; 4 Department of Biochemistry and Genetics, School of Medicine, Lorestan University of Medical Sciences, Khor-ramabad, Iran; 5 Department of Internal Medicine, Shahid Beheshti Hospital, Babol University of Medical Sciences, Babol, Iran

**Keywords:** pomegranate seed oil, polycystic ovary syndrome, effects, epidemiology, health, linolenic acid, linoleic acid

## Abstract

Polycystic ovary syndrome is the most common endocrine disorder in women. Today, medicinal plants have been considered by women, especially in the reproductive and pregnancy ages. Multiple drug treatments and the length of the treatment period often lead to incomplete treatment by patients. Therefore, due to the side effects of chemical drugs, this study was conducted to assess investigate the effect of pomegranate seed oil on polycystic ovary syndrome. The prevalence of polycystic ovary syndrome is increasing by 15 to 20% and clinically includes oligomenorrhea or amenorrhea, hirsutism, and often infertility. Databases such as Cochran library, Medline, PubMed, SID, and Science Direct were used to access the related articles. To collect the required information, first, the articles that had one of the keywords of medicinal plants, polycystic ovary syndrome, plant, pomegranate extract, and menstrual irregularities in their text were searched in databases. All studies from 1985 to 2021 are included in the study. Conjugated linolenic acid (CLN) is a group of geometric and positional isomers of linolenic acid in which double bonds are conjugated. CLN has been reported to have a very strong cytotoxic effect on tissue tumor cells in the body, preventing cancer, reducing the accumulation of triacylglycerol in the liver, polycystic ovary syndrome, and LDL cholesterol in the blood. So far, seven CLN isomers have been identified, including ponic acid in pomegranate seed oil. Conjugated linoleic acid (CLA) is a group of situational and geometric isomers of linoleic acid in which double bonds are conjugated. The positive effects of the two main CLA isomers (cis-9, trans-11, and trans-10, cis-12) include inhibiting the growth of cancer, reducing the risk of atherosclerosis, and reducing body fat.

## INTRODUCTION

Polycystic ovary syndrome (PCOS) is the most common endocrine disorder and metabolic heterogeneity in women of childbearing age. It involves various factors involved influenced by environmental factors such as eating habits, lifestyle, and social status (Beydoun *et al*., 2009). The most important feature of this disorder is the presence of large ovaries containing a large number of small cysts that are located in the outer layer of each ovary. In this syndrome, the levels of female hormones (such as estrogen, progesterone) become unbalanced and cause an increase in male hormones (androgens) and insulin. This causes symptoms such as menstrual irregularities, hair loss, acne, infertility, and obesity (Goodarzi *et al*., 2012; Wang *et al*., 2011).

Epidemiological studies show that the prevalence of PCOS varies from 2.2-26% in different countries (March *et al*., 2010; Tehrani *et al*., 2011). In Brazil, the prevalence of PCOS in women aged 18-45 years was estimated at 8.5% (Gabrielli & Aquino, 2012) and in Italy in adolescents aged 18 years 32% (Franceschi *et al*., 2010). Various treatments have been proposed for polycystic ovary syndrome; such as lifestyle changes, surgery, and the use of chemical drugs that today the use of medicinal plants, has been significantly considered by researchers due to the side effects and risks of chemical drugs, especially in mothers of childbearing age (Ortmann *et al*., 2002). Pomegranate is one of the fruits that is widely consumed all over the world and has been of considerable importance in the pharmaceutical and food industries.

Pomegranate is a fruit-bearing deciduous shrub or small Asian tree with the scientific name of Punica granatum, which reaches a height of 5 to 8 meters. This plant is native to Iran and northern India and has long been cultivated in the Mediterranean region. This plant is currently widely cultivated throughout Armenia, Iran, India, South Asia, East India, and tropical Africa. Pomegranate is a drought-tolerant plant that can grow in arid areas with the Mediterranean climate, winter rainfall, or summer rainy climate. In Iran, pomegranates are grown mainly in desert areas with hot and dry summers, scorching suns, relatively cold winters, and salty soil and water; this wide range of adaptation is one of the desirable characteristics of pomegranate ecophysiology. Pomegranate fruit has a thin leathery skin that changes color from red to pinkish yellow. The kernels are edible and are covered with a fleshy, transparent, and white to a red color called aryl with a sour-sweet taste. Pomegranate kernels make an average of 143-37 grams per kilogram of pomegranate fruit, kernels are rich in oil and about 12-18% of it is oil (Radjabian *et al*., 2008). The pomegranate seed oil )PSO) contains a variety of fatty acids, about 80% of which are 18-carbon fatty acids with three conjugated double bonds (punic acid). Studies have shown that ponic acid is physiologically much stronger than Conjugated linoleic acid )CLA( (Mukherjee *et al*., 2002).

There are very few reports on the pharmacological effects of PSO. A study by Nikseresht *et al*. (2015) showed that PSO had a positive effect on the fertilization power of male rats. These effects can be helpful for infertility. PSO supplementation may reduce oxidative stress in sperm and increase the pregnancy rate in women. In one study, pomegranate seed extract probably played an important role in reducing postpartum hemorrhage in mice (Hossein *et al*., 2015). A grape seed extract has probably increased the number of ovarian follicles and the formation of corpus luteum in the polycystic ovary due to its antioxidant and anti-inflammatory properties (Salmabadi *et al*., 2017).

In a study by Arao *et al*. (2004), the effect of punic acid-rich PSO on fat metabolism in high-cholesterol mice was investigated. The results showed that feeding mice with PSO significantly reduced the accumulation of triglycerides and monounsaturated fatty acids in their liver. PSO prevents breast cancer due to its phytoestrogens and is also used in medicinal products effective in preventing skin aging.

Pomegranate seed oil and juice is a synthetic antioxidant with carcinogenic properties. For this reason, in recent years, much attention has been paid to the replacement of natural antioxidants, especially in plant sources. Due to the lack of data on the possible effect of pomegranate seed water and oil consumption on patients with polycystic ovary syndrome, this study aimed to investigate the effect of pomegranate seed oil on human health.

## METHODS

A complete the databases searched for in those articles were “Google Scholar”, “SID”, “Scopus”, “PubMed”, “Science Direct”, and “ISI” search engines. The search was done for articles published that included the search the effect of pomegranate seed oil on human health in their title. This study focused on published articles papers from 1985 to 2021.

## RESULTS AND DISCUSSION

### Lipids and their types

Lipids are a wide range of substances that are generally soluble in organic solvents and insoluble in water. The terms fat and oil are two functional equivalents for lipids that are sometimes used interchangeably. But in general, fat can be considered a type of lipid that is solid at room temperature, while the term oil is used for liquid lipids at room temperature (Nichols & Sanderson, 2002). In the human body, saturated and monounsaturated fatty acids are synthesized from acetyl coenzyme A or obtained from diets. In contrast, some fatty acids, such as linoleic acid and linolenic acid, are not synthesized by humans due to a lack of necessary enzymes and must be supplied through food, which is why these compounds are called essential fatty acids (EFA). These fatty acids are part of omega-3 and omega-6 fatty acids, which make up one-third of intracellular fatty acids. Essential fatty acids are also involved in the structure of phospholipids, which play a role in cellular structure, and when phospholipids are deficient, energy metabolism is disrupted. Essential fatty acids also lower cholesterol, so their presence or the ratio of essential fatty acids to saturated fatty acids is a factor in lowering blood cholesterol (Sikorski & Kolakowska, 2002). A proper balance between the number of lipids in the diet and its nutritional quality is very important in maintaining human health. Today, it has been proven that some fats and oils in the human body have beneficial properties and in addition to having nutritional value, they can also have positive effects on human health. These compounds are called functional Lipid, Nutritional Lipid, It is called Medical Lipid or Pharmaceutical Lipid. For this reason, the food industry, considering the increasing awareness of people about nutrition and health issues, is trying to introduce such products to the consumer and meet the needs of the market by producing such products (Adhikari *et al*., 2010).

### Conjugated Linolenic Acid (CLN)

A conjugated linolenic acid is a group of geometric and positional isomers of linolenic acid in which double bonds are conjugated. CLN isomers are found in the oils of certain plant seeds and often as the major fatty acid.

So far, seven CLN isomers have been identified, including Eleostearic Acid (ESA-α) in Bitter Melon seed oil, Punicic Acid (PA) in pomegranate seed oil, Catalpic Acid in Jacaric Acid seed oil, and Jacaranda mimosifolia in pomegranate seed oil (Adhikari *et al*., 2010).

### Effects of CLN on human health

#### The effect of pomegranate seed oil on polycystic ovary syndrome

PCOS is the most common endocrine or endocrine disorder in women and the cause of infertility due to ovulation, which affects different aspects of people’s lives due to different symptoms. This syndrome occurs in 10-15% of girls and women. Manifestations of this syndrome include hirsutism, hyperandrogenism, acne, endometrial thinning, menstrual disorders, and obesity (Makker *et al*., 2012; Moradi & Darvishi, 2009; Yildirim *et al*., 2003).

In recent years, many studies were done on the use of herbal medicines in the treatment of polycystic ovary syndrome. Changes in sex hormone levels are important in polycystic ovary syndrome, and serum concentrations of testosterone and androstenedione are 50 to 150% higher in women with PCOS than in normal women (Attarzadeh *et al*., 2012). In a study by Hossein *et al*. (2015) the use of pomegranate extract improved changes in female sex hormones in patients with PCOS. Therefore, it was suggested that this extract be used to reduce the symptoms of PCOS.

Esmaeilinezhad *et al*. (2019) examined the effect of synbiotic pomegranate juice on blood glucose indices, sex hormone profile, and anthropometric indices in polycystic ovary syndrome, the results showed that testosterone levels, BMI, and weight were significantly reduced. Shamsi *et al*. (2016) showed that Glycyrrhiza glabra extract reduces serum levels of testosterone and estrogen in the study of the effect of glycyrrhizic acid on ovarian tissue follicles in mice with polycystic ovaries.

Serological changes of curcumin were also observed in the form of increased FSH and progesterone, decreased LH, estradiol, and testosterone (Nabiuni *et al*., 2015) by 18% and 23% (and reduced progesterone (Mokhtari *et al*., 2014). Phenolic compounds similar to pomegranate extract show positive effects in improving the complications of PCOS (Milewicz *et al*., 1993). Evaluation of the role of possible improvement of pomegranate juice extracts on endometrial damage in a rat model with endometrial histological changes through antioxidant, anti-inflammatory, and anti-fibrotic effects, due to its polyphenol content (Ibrahim *et al*., 2021). Study by Fozalaee *et al*. (2015) and Pahlevani *et al*. (2016) examined the effect of fennel extract and Stachys lavandulifolia on uterine tissue structure in mice with PCOS, respectively. Kouchesfahani *et al*. (2010) in a study showed that honey bee venom with antioxidant and anti-inflammatory effects in polycystic ovary affected rats increased the number of small follicles and the appearance of corpus luteum in the ovary.

Free radicals are responsible for membrane lipid peroxidation, decreased sperm motility, and sperm inability to fertilize eggs (Gil-Guzman *et al*., 2001). Pomegranate juice contains vitamins C and E, which play an important role in targeting free radicals (Sönmez *et al*., 2005; 2007). The effect of pomegranate extract on sperm parameters, fertility potential in mice, and improving sperm parameters are effective and its administration is recommended to improve the sperm status of infertile men because it is herbal and available (Amini Rad *et al*., 2009).

### Anti-cancer properties

According to reports, CLN has a very strong cytotoxic effect on tumor tissue cells in the body and prevents colon and skin cancer in mice and a rapid increase in breast cancer cells in humans (Eikani *et al*., 2012). Kohno *et al*. (2004) fed rats with diets of different concentrations of pomegranate seed oil, studied the effects of conjugated linolenic acid on the prevention of colon cancer and found that pomegranate seed oil, due to its rich CLN fatty acid content, could Prevent the formation of colon cancer cells.

### Anti-atherosclerosis

Arao *et al*. (2004) reported that linolenic acid affected on the indicated that by feeding a diet enriched with pomegranate seed oil in rats, they observed positive effects of ponic acid on liver triglyceride levels.

### Anti-atherosclerotic properties

Cardiovascular disease caused by high blood lipid levels is a very common problem that has led to many deaths. Studies show that changing lifestyle habits such as eating habits, exercising, and avoiding smoking can help prevent these diseases (Marcus *et al*., 1999; Ross, 2000). Diet plays a major role in reducing the risk of cardiovascular disease, and extensive research on specific foods and components has shown that improving the incidence of serum lipids can reduce the incidence of these diseases (Duthie & Brown, 1994; Kannel, 1985). Radjabian *et al*. (2008) showed that the progression of atherosclerosis in the aorta of rabbits in the group treated with pomegranate seed oil was significantly reduced compared to the control group. Mukherjee *et al.* (2002) examined the effect of different concentrations of Punicic Acid (0.6, 2.1, 4.2%) on laboratory mice. Finally, a significant reduction in plasma cholesterol and LDL levels was observed in the group treated with 2.4% Punicic Acid.

### Conjugated Linoleic Acid

CLA is a general term for all positional and geometric isomers of linoleic acid conjugated DNA. Linoleic acid, or 9-cis, 12-cis octoic acid, is an 18-carbon double-bonded fatty acid in positions 9 and 12. All known physiological effects of CLA are related to only the two major isomers of CLA, t11 and t10, c12. The main sources of CLA are naturally ruminant animal fats. Milk and dairy products have the highest amount of CLA. The CLA levels in some foods are shown in Table 1. Among meat products, ruminant meat has higher levels of CLA than non-ruminant meat. The highest amount of CLA in meat products is found in mutton. Vegetable oils and margarine also contain a very small amount of CLA (0.1-0.5 mg per gram of fat), which is the result of industrial processes such as oil refining (especially the roasting step) and the hydrogenation process. In addition, CLA can be produced in oil due to high heat processes, for example, sunflower oil after use for frying contains 0.5 grams of CLA per 100 grams of oil (Wahle *et al*., 2004; Gnädig *et al*., 2003).

**Table 1:** Conjugated linoleic acid (CLA) levels in some foods (Wahle *et al*., 2004).

Food	CLA rate %	Percentage of isomer c9, t11
Milk	5.5	92
Butter	4.7	88
Sour cream	4.6	90
Yogurt	4.8	84
Ice cream	3.6	86
Cheddar cheese	3.6	93
Mozzarella cheese	4.9	95
Fresh beef	3.4	85
Lamb meat	5.6	92
Veal	2.7	84
Chicken	0.9	84
Turkey meat	2.5	76
Sunflower oil	0.4	38
Safflower oil	0.7	44
Canola oil	0.5	44
Corn oil	0.2	39

### Effects of linoleic acid on human health Reduce body fat

Today, obesity is an important factor in the development of heart disease, diabetes, and high blood pressure. The first report on CLA’s ability to reduce body fat in a rat treatment was published in 1995. In that study, it was found that using CLA at a rate of 0.5% in the diet reduces body fat and increases the amount of protein and water. In 1999, it was discovered that this CLA effect was related to the isomers t10, c12 (Wahle *et al*., 2004; Park & Pariza, 2007). In another study, it was found that the supplementation of CLA isomers (concentration of both isomers c9, t11 and t10, c12 in the mixture was 50%) by 3 grams per day, the plasma triglyceride concentration is rapidly reduced. The ability of CLA to alter lipid metabolism depends on factors such as increased lipolysis rate and fatty acid oxidation and decreased cellular fatty acid uptake.

The effect of CLA on reducing body fat in humans was less than in mice, which could be due to the following reasons: (Franceschi *et al*., 2010) The dose of CLA used in humans was much lower than the dose used in mice. Mice were fed a diet containing (w / w) 0.5% CLA, which is equivalent to 56 grams of CLA per 70 kg individual. While the dose of CLA for humans in various studies was between 0.7-6.8 grams per day, which is much lower than the estimated 56 grams (Goodarzi *et al*., 2012). Different responses to CLA in humans and mice may be due to differences in their fat metabolism. In general, to accurately identify the mechanism of action of CLA in reducing body fat and its effects, more studies and research are needed.

### Antioxidant properties

Changing the profile of fatty acids in cancer cells and interfering with the production of eicosanoids.

The effect on the growth and increase of certain types of cancer cells and Apoptosis (Ha *et al*., 1990).

### Anti-atherosclerotic property

Oxidative stress is one of the main causes of atherosclerosis. LDL oxidation is a well-known mechanism in this regard. Evidence suggests that the disease can be cured or controlled with antioxidants. As mentioned, the antioxidant properties of CLA have been proven in many studies (Flintoff-Dye & Omaye, 2005). Kostogrys & Pisulewski (2010) investigated the effect of CLA oil on a high-fructose diet in Wistar rats and concluded that CLA in this type of diet reduces plasma triglycerides and LDL.
